# Assessing mechanisms for microbial taxa and community dynamics using process models

**DOI:** 10.1002/mlf2.12076

**Published:** 2023-09-12

**Authors:** Linwei Wu, Yunfeng Yang, Daliang Ning, Qun Gao, Huaqun Yin, Naija Xiao, Benjamin Y. Zhou, Si Chen, Qiang He, Jizhong Zhou

**Affiliations:** ^1^ Institute of Ecology, Key Laboratory for Earth Surface Processes of the Ministry of Education, College of Urban and Environmental Sciences Peking University Beijing China; ^2^ Institute for Environmental Genomics University of Oklahoma Norman OK USA; ^3^ Department of Microbiology and Plant Biology University of Oklahoma Norman OK USA; ^4^ State Key Joint Laboratory of Environment Simulation and Pollution Control, School of Environment Tsinghua University Beijing China; ^5^ School of Minerals Processing and Bioengineering Central South University Changsha China; ^6^ Department of Mathematics, Lunt Hall Northwestern University Evanston Illinois USA; ^7^ Department of Civil and Environmental Engineering The University of Tennessee Knoxville Tennessee USA; ^8^ Institute for a Secure and Sustainable Environment The University of Tennessee Knoxville Tennessee USA; ^9^ School of Civil Engineering and Environmental Sciences University of Oklahoma Norman Oklahoma USA; ^10^ Earth and Environmental Sciences, Lawrence Berkeley National Laboratory Berkeley California USA; ^11^ School of Computer Science University of Oklahoma Norman OK USA

**Keywords:** community assembly mechanisms, consumer–resource model, neutral model, species dynamics

## Abstract

Disentangling the assembly mechanisms controlling community composition, structure, distribution, functions, and dynamics is a central issue in ecology. Although various approaches have been proposed to examine community assembly mechanisms, quantitative characterization is challenging, particularly in microbial ecology. Here, we present a novel approach for quantitatively delineating community assembly mechanisms by combining the consumer–resource model with a neutral model in stochastic differential equations. Using time‐series data from anaerobic bioreactors that target microbial 16S rRNA genes, we tested the applicability of three ecological models: the consumer–resource model, the neutral model, and the combined model. Our results revealed that model performances varied substantially as a function of population abundance and/or process conditions. The combined model performed best for abundant taxa in the treatment bioreactors where process conditions were manipulated. In contrast, the neutral model showed the best performance for rare taxa. Our analysis further indicated that immigration rates decreased with taxa abundance and competitions between taxa were strongly correlated with phylogeny, but within a certain phylogenetic distance only. The determinism underlying taxa and community dynamics were quantitatively assessed, showing greater determinism in the treatment bioreactors that aligned with the subsequent abnormal system functioning. Given its mechanistic basis, the framework developed here is expected to be potentially applicable beyond microbial ecology.

## Impact statement

One fundamental goal in microbial ecology is to predict how microbial diversity is changed across space and time. Although spatial patterns of microbial communities have been recently intensively examined, our understanding of microbial temporal dynamics is rudimentary, primarily due to the lack of appropriate experimental data and theoretical framework. By reconciling niche and neutral perspectives, this study developed a novel process model‐based framework to effectively encapsulate microbial species temporal dynamics, which is powerful for quantitatively assessing the assembly mechanisms underlying microbial community dynamics. This study represents a significant advance in explaining microbial temporal dynamics toward predictive microbial community ecology.

## INTRODUCTION

Microorganisms are the most diverse group of life on Earth and play critical roles in global biogeochemical cycling of carbon, nitrogen, phosphorus, sulfur, and various other elements. It is well known that microbial diversity is extremely high across various habitats^1^, ^2^, ^3^. One of the fundamental goals in microbial ecology is to determine how such extremely high microbial biodiversity is generated and maintained across space and time^4^. Two types of ecological processes (deterministic vs. stochastic) are influential for explaining the processes of assembling individual taxa into a local community. Niche‐based theory assumes that deterministic processes, such as differences in taxonomic and functional traits, interspecies interactions (e.g., competition, predation, and mutualisms), and abiotic filtering (e.g., temperature, pH), are responsible for local community compositions^5^, ^6^. In contrast, neutral theory proposes that all species are ecologically equivalent; thus, immigration and ecological drift of stochastic birth and death shape the diversity and composition of local communities regardless of species traits^7^. Although both deterministic and stochastic processes are believed to play key roles in shaping community diversity, their relative importance is still hotly debated^6^, ^7^, ^8^, ^9^, ^10^, ^11^, particularly in microbial ecology^4^, ^12^, ^13^, ^14^. It is thus critical to quantify the degree to which deterministic or stochastic processes impact community assembly, in order to effectively influence or manipulate microbial communities for designed functions^4^, ^14^.

Several major approaches have been used to infer community assembly mechanisms, such as multivariate analysis, null modeling analysis, and ecological theory‐based process models (i.e., niche and neutral models)^4^, ^15^. Compared to the multivariate and null model‐based statistical approaches, the ecological theory (niche vs. neutral)‐based process model approach is more attractive because it allows mechanistic predictions of community dynamic behavior. One of the most widely used niche models is Lotka–Volterra competition^16^, ^17^, which describes the dynamics of individual taxa as a function of growth rate and inter‐species interaction. However, this direct effect is rarely analyzed in nature, and it is challenging to measure the competition coefficients experimentally^17^, ^18^. Such parameter‐rich models are particularly intractable for studying microbial communities that typically show high diversity^19^, ^20^, ^21^, ^22^. An alternative to the generalized Lotka–Volterra model is the consumer–resource model, which describes the dynamics of individual taxa as a function of the availability of resources. This model assumes that species interact only through competition for a few limiting resources^23^, ^24^, which greatly reduces the number of required parameter from the square of the taxon number (pairwise species interactions) to the number of resources, and hence it is parsimonious for complex systems such as microbial communities^25^. Recently, resource‐related models have been successfully used for modeling microbial community diversity dynamics^26^, ^27^.

Neutral models have also been successful in explaining some of the most widely studied patterns in community ecology, such as abundance distribution^28^, rank‐abundance distribution,^13^ and frequency‐abundance distribution of individual taxa^12^. However, most studies have focused on community‐level predictions at one time point^29^, ^30^, ^31^, ^32^, ^33^, but rarely examined the dynamic behavior of individual populations from neutral perspectives^13^. This is an important knowledge gap to fill because temporal dynamic behavior is critical for understanding multispecies coexistence^6^ and functional stability^34^. Also, because both niche and neutral mechanisms play key roles in community assembly^35^, several studies attempted to develop unified models to reconcile both mechanisms^6^, ^8^, ^9^, ^10^. However, such theoretical models are rarely applied to actual ecological data owing to mathematical challenges^36^, ^37^. Recently, a stochastic differential equation (SDE)‐based model that consolidates niche and neutral processes has been developed to simulate the dynamics of individual microbial taxa^13^, ^36^. Rooted on the framework of a neutral model, this SDE model considers the niche effect by incorporating an advantage term as a linear function of various environmental variables^13^. However, this SDE model does not account for biotic interactions such as competition.

In this study, we developed a novel process model‐based framework to quantitatively infer assembly mechanisms by integrating niche and neutral theory‐based models for community dynamics. Specifically, we first developed an SDE‐based combined model by incorporating consumer–resource interactions, immigration, and drift. We then compared this new model with the consumer–resource model and neutral model, for the ability to capture the temporal dynamics of individual taxa in anaerobic bioreactors. We estimated ecologically relevant model parameters such as the immigration rate and competition strength, and inferred the relative importance of stochastic versus deterministic processes in driving community dynamics. We applied this framework to analyze time‐series data from anaerobic bioreactors under stable or disturbed process conditions. Our results indicated that it provides a robust, reliable process model‐based tool for assessing assembly mechanisms controlling taxa and community dynamics.

## RESULTS

### Overview of modeling framework

**Figure 1:**
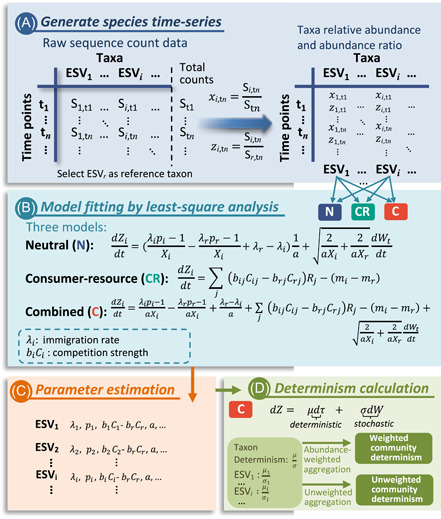
Overview of the framework. (A) The raw sequence data are processed to generate the time series of taxa relative abundances and the abundance ratio of focal taxon to the reference taxon. (B) The neutral, consumer–resource, and combined model are fitted using the least‐square methods for each taxon. (C) Key parameters can be estimated from modeling. (D) The taxa and community determinism are assessed based on the estimated parameters of the combined model. ESV, exact sequence variant.

To assess the mechanisms controlling community dynamics, raw time‐series sequence data are first processed to generate relative abundances of individual taxa represented as exact sequence variants (ESVs) (Figure 1A). The reference taxon is chosen as the one with the top frequency and relative abundance, and the ratio of taxa abundance to the abundance of the reference taxon is then calculated for each taxon. The observed time‐series data of each taxon are then fitted with the neutral, consumer–resource, and combined models (Figure 1B). The performances of the three models are compared according to the Akaike information criteria (AIC) values, aiming to reveal potential mechanisms driving the dynamics of individual taxa. Ecologically important parameters, such as $\lambda_{i}$ (the rate of migration from the metacommunity into the local community) and ${b_{i}C}_{i}-{b_{r}C}_{r}$ (relative competition strength to the resource), are estimated using the least‐square method for model fitting (Figure 1C). Finally, the determinism for taxa and community dynamics are assessed based on the SDEs of the combined model (Figure 1D), as the SDEs comprise the deterministic and stochastic part. It is noted that, while the immigration is generally considered as a stochastic process^15^, it is included in the deterministic part of the SDEs (Equations 6, 9, and 11). In fact, the immigration process acts as a restoring force that makes the relative abundance return to its mean value when there is a deviation between the current relative abundance and the mean relative abundance.

### Model performances on taxon dynamics

To illustrate how the process model‐based framework (Figure 1) is applied to microbial time‐series data, we collected longitudinal data from two contrasting sets of anaerobic bioreactors, each with three replicates (Figure S1): There were a total of 53‐time points from the control bioreactors in which stable process conditions were maintained over 500 days and 11‐time points from the treatment bioreactors over 100 days during which the resource levels were incrementally increased until process conditions deteriorated to an ultimate collapse. A total of 6799 microbial taxa, represented by ESVs, were detected, which were present in at least one sample in control or treatment bioreactors. Further, models were fitted using the least‐squares method for each taxon under control or treatment conditions, requiring the taxon to present in at least six time points (e.g., a taxon present in at least six out of 53‐time points in bioreactor C1). Specifically, we combined the time series of the triplicate treatment bioreactors together to improve the reliability of model fitting (see Materials and Methods section for details), and fitted the models on taxa that were present in at least six out of 33 time points in treatment bioreactors. In addition, the mean relative abundance of each taxon in control or treatment bioreactors was calculated, based on which taxa were classified into three groups: the abundant taxa (mean relative abundance >0.1%), the moderate taxa (mean relative abundance between 0.01% and 0.1%), and the rare taxa (mean relative abundance <0.01%) (Table S1).

**Figure 2:**
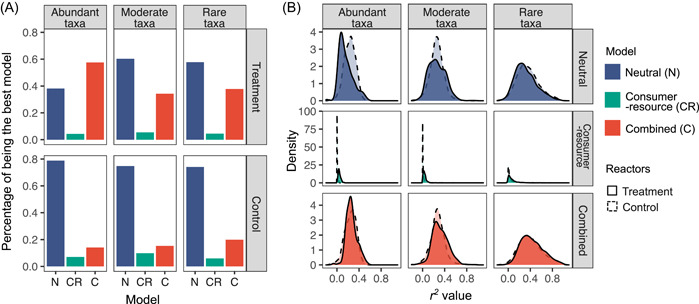
Model fitting on microbial taxa in control bioreactors with stable substrate feeding and treatment bioreactors with incremental substrate feeding. (A) Percentages of the neutral model (N), the consumer–resource model (CR), and the combined model (C) being the best model describing taxon dynamics. For each taxon, we fitted the three models, and the best model for that taxon was determined as the one with the lowest Akaike information criteria (AIC) value. Three groups of taxa were classified by mean relative abundance, with mean relative abundance <0.01% for rare taxa, from 0.01% to 0.1% for moderate taxa, and >0.1% for abundant taxa. (B) Distribution of *r* ^2^ values of the three models.

To identify the mechanisms driving the dynamics of individual taxon, the relative performances of the three models were compared based on AIC values. In the treatment bioreactors, the combined model had the best fit for 58% of the abundant taxa (Figure 2A), suggesting that most abundant taxa were driven by both stochastic drift and deterministic immigration and competition. In contrast, the neutral model had the best fit for 38% of the abundant taxa, and the consumer–resource model had the best fit for only 4% of the abundant taxa. For rare taxa, 58% of them in the treatment bioreactors found best fit with the neutral model, suggesting that rare taxa were mainly shaped by immigration and drift. The importance of neutral processes was even more conspicuous in the control bioreactors, since the neutral model had the best fit for 79% of all abundant taxa and 74% of rare taxa. Therefore, neutral processes of immigration and drift were identified to drive the dynamics of the majority of rare taxa, particularly in the control bioreactors. When examining the model performance for the entire community, the neutral model had the best fit for most taxa in both the control (75% of all taxa) and treatment bioreactors (57% of all taxa) (Figure S2A), which was expected as the rare taxa contributed to the majority of the taxa number (Table S1).

Model performance was further examined across the major phyla. The neutral model was the best for most rare taxa in both treatment and control bioreactors regardless of phylogenetic relationships (Figure S3), while the combined model performed better than the other two models for the abundant taxa in treatment bioreactors for five out of the top seven phyla such as *Firmicutes* and *Bacteroidetes* (Figure S3). These results suggested that model performance was largely unrelated to microbial phylogeny.

Because the combined model includes both the neutral and consumer–resource interaction terms, the *r* ^2^ values from the least‐square fitting are almost always the largest for the combined model (Figure 2B). On average, the combined model can explain 36% ± 20% (mean ± SD) of the variations in taxon dynamics based on the *r* ^2^ values, while the neutral model can explain 31% ± 19% and the consumer–resource model can only explain 4% ± 8% of the variations (Figure S2B). Regarding the ability to represent taxon dynamics under different treatment conditions, the neutral model could explain more variations of the abundant taxa in the control than the treatment bioreactors (mean *r* ^2^ value: 22% v.s. 16%; *P* < 0.0001 by a two‐tailed *t*‐test) (Figure 2B). It also performed better on the rare taxa in the control than the treatment bioreactors (mean *r* ^2^ value: 36% vs. 32%; *P* < 0.0001 by a two‐tailed *t*‐test). In contrast, the consumer–resource model or the combined model was able to represent taxon dynamics in the treatment bioreactors better than those in the control bioreactors, as the mean *r* ^2^ values were significantly higher in the treatment than the control bioreactors for abundant, moderate, and rare taxa (*P* < 0.02 by two‐tailed *t*‐test). Therefore, the relative performance of these three models is dependent on taxa abundance and process conditions in the ecosystem of interest.

### Competition strengths among different taxa

Ecologically important parameters such as ${b_{i}C}_{i}-{b_{r}C}_{r}$ reflecting the relative competition strength can be estimated with relative taxon abundance data at discrete time points, based on the consumer–resource model or the combined model. Considering the better performance of the combined model than the consumer–resource model, here, the parameters were estimated based on the combined model to enable the comparison across taxa, which are summarized in Table S2. The top three most competitive taxa in the treatment bioreactors were identified to be associated with the genera *Ornithinicoccus*, unclassified *Ruminococcaceae* and *Gottschalkia*, suggesting them as strong competitors for the organic substrates.

**Figure 3:**
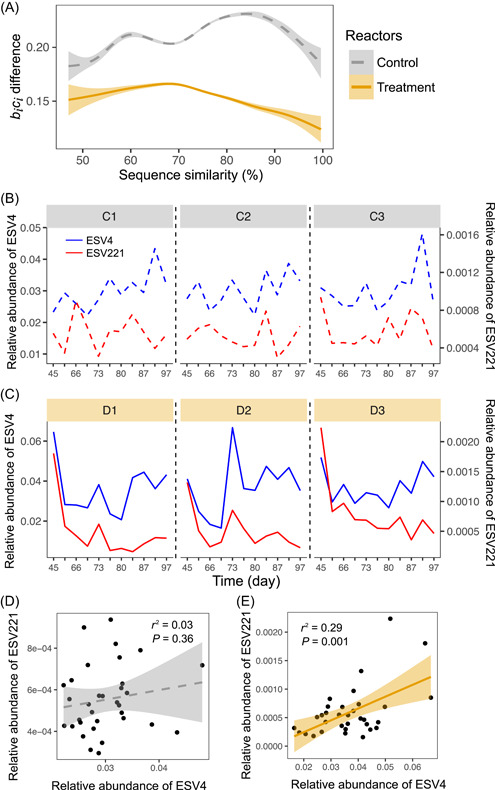
Relationship between ESVs' sequence similarity and the difference of estimated $b_{i}C_{i}$ representing the competition strength for resource. (A) Smoothed lines showing the mean difference in ${b_{i}C}_{i}$ at different sequence similarity levels between ESVs. The shaded area represents the 95% confidence interval. (B) The time series of two taxa in the control reactors. The two taxa, ESV4 and ESV221, were from genus T78 of the family Anaerolineaceae, and they were 98.8% similar in 16S sequences. (C) Time series of ESV4 and ESV221 in the treatment reactors showing consistent fluctuations of their relative abundances. (D, E) Correlation between ESV4 and ESV221 in control (D) and treatment (E) reactors. C1, C2, and C3 represent the control bioreactors; D1, D2, and D3 represent the treatment bioreactors.

We were curious whether phylogenetically closely related taxa are more likely to have similar competition strengths. Thus, we examined the relationship between taxa phylogeny and the estimated relative competition strength. When the sequence similarity between taxa was larger than 70%, the difference in ${b_{i}C}_{i}$ had a significant negative correlation with sequence similarity in the treatment bioreactors (Spearman's *ρ* = −0.04, *P* < 0.0001) (Figure 3A), suggesting that closely related microbial taxa had similar competition strengths (i.e., phylogenetic signal) when resource levels were altered. The negative correlation between competition strength difference and sequence similarity robustly held under even higher sequence similarity (Spearman's *ρ* = −0.04, *P* < 0.0001 for sequence similarity >80% and Spearman's *ρ* = −0.07, *P* = 0.003 for sequence similarity >90%). However, this negative correlation did not hold when the sequence similarity of the 16S rRNA gene was less than 70% (Spearman's *ρ* = 0.03 for treatment bioreactors). For control bioreactors, the negative correlation between sequence similarity and the difference in ${b_{i}C}_{i}$ was observed when sequence similarity was larger than 85% (Spearman's *ρ* = −0.06, *P* < 0.0001) but not below that threshold (Figure 3A). Therefore, the phylogenetic signal of resource competition strengths is relevant only within certain phylogenetic distances. It is also noted that, although significant, the correlations were weak (absolute Spearman's *ρ* < 0.1), suggesting that phylogeny could only explain a minor proportion of variations in taxa resource competition strengths.

Since the mean ${b_{i}C}_{i}\;$difference of microbial taxa was substantially larger in control bioreactors (0.21 ± 0.19, mean ± SD) than that in treatment bioreactors (0.16 ± 0.14, mean ± SD), microbial responses to resource levels were more predictable in the treatment bioreactors, where changes in resource levels could lead to greater environmental selection. As a result, temporal dynamics of closely related ESVs was more similar in the treatment bioreactors than the control bioreactors. For example, ESV4 and ESV 221, which are 98.8% similar in sequence, belong to the same genus T78 of family *Anaerolineaceae*. The temporal dynamics of their relative abundance were not correlated (Pearson's *r* = 0.17, *P* = 0.36) in the control bioreactors (Figure 3B,D) but significantly correlated (Pearson's *r* = 0.54, *P* = 0.001) in the treatment bioreactors (Figure 3C,E).

### Negative correlation between immigration rates and taxa abundances

**Figure 4:**
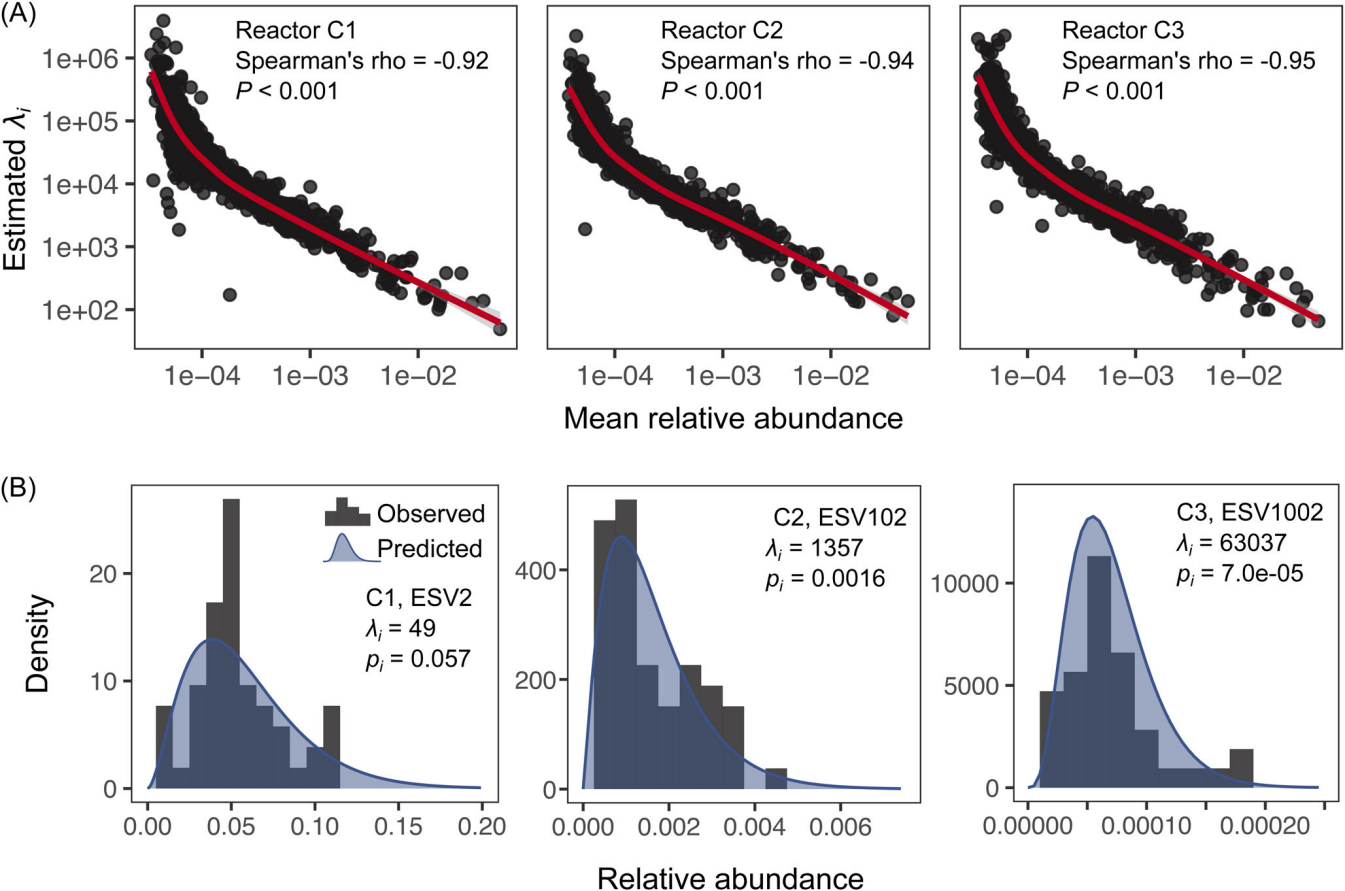
Testing the neutral model on species time series in control bioreactors. (A) Estimated *λ*_*i*_ from the neutral model versus the mean relative abundance of all taxa in each reactor. (B) Prediction of the neutral model on the distribution of the relative abundances of several exemplified ESVs. When the local community size was large, the relative abundance of a specific taxon followed a beta distribution under neutral scenarios, whose shape was determined by parameters *λ*_*i*_ and *p*_*i*_ (the relative abundance of this taxon in the source community)^12^. The gray histograms represent the observed value, and the dark blue shadow represents the model predictions using the parameters *λ*_*i*_ and *p*_*i*_ calibrated from the time series.

The neutral model presented the best fit for most taxa in the control bioreactors (Figure 2A). We further examined how the estimated *λ*_*i*_, which represents the immigration rates, varied across all taxa. The estimated relative immigration rates were similar for the same ESVs across triplicate bioreactors but highly different among various taxa, spanning differences of up to 10^4^ folds. The estimated values of *λ*_*i*_ were negatively and significantly (Spearman's *ρ* = −0.95 to −0.92, *P* < 0.0001) correlated with the average relative abundances of ESVs (Figure 4A). Furthermore, the estimated *λ*_*i*_ values were highly variable within each phylum because they were negatively dependent on taxa abundance (Figure S4), suggesting that the estimated immigration rates were related to abundance but not phylogeny.

The probability density distribution of individual taxon abundance under equilibrium can be derived for the neutral model^12^. This abundance distribution is not possible for the consumer–resource or the combined model because taxon dynamics is dependent on the resource variable in these models. The probability density distributions of the relative abundances of an ESV can be predicted by *λ*_*i*_ and *p*_*i*_ (the relative abundance of that ESV in the source community) in the neutral model, which were shown to follow a beta distribution^12^. Exemplified by the distributions of relative abundances for several representative ESVs ranging from abundant to rare ones in the control bioreactors, the beta distributions predicted the dynamics of ESVs well, with much higher *λ*_*i*_ values for the rarer taxa (Figures 4B and S5). These results suggested that the neutral model could be used to predict the range of fluctuation for each microbial taxon under equilibrium, which may be valuable for assessing the boundaries of population abundance in a stable microbial community.

### Higher determinism in the treatment bioreactors

**Figure 5:**
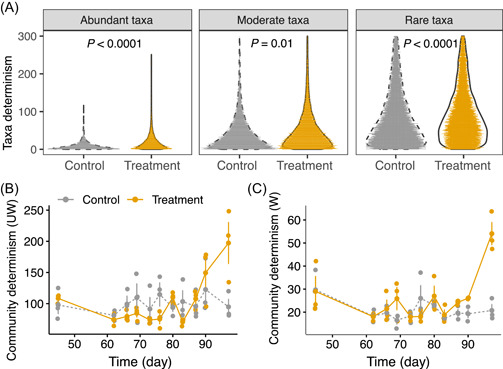
Species‐level and community‐level determinism. (A) Predicted determinism across taxa under control and treatment bioreactors. (B, C) Comparisons of the predicted unweighted (B) and weighted (C) community‐level determinism between the control and treatment reactors. The lines represent the mean determinism of the three replicated control or treatment bioreactors, and the error bars represent the standard deviations.

The determinism of taxa at certain time points was calculated based on the parameters estimated of the combined model using the above‐mentioned approach (Figure 1). Interestingly, taxa determinism showed a significant negative correlation with the mean relative abundance of taxa in both control (Spearman's *ρ* = −0.53, *P* < 0.0001) and treatment bioreactors (Spearman's *ρ* = −0.55, *P* < 0.0001) of rare and abundant taxa, suggesting that rare taxa tended to be more predictable than abundant taxa. Further, the mean taxa determinism was higher in treatment than control bioreactors for abundant (mean determinism: 16 vs. 13; *P* < 0.0001 by a two‐tailed *t*‐test), moderate (mean determinism: 57 vs. 54; *P* = 0.01 by a two‐tailed *t*‐test), and rare taxa (mean determinism: 196 vs. 152; *P* < 0.0001 by a two‐tailed *t*‐test) (Figure 5A).

The community‐level determinism was further derived by aggregating the determinism of co‐occurring taxa within the community. The abundance‐weighted determinism and unweighted community determinism were not different between the control and treatment bioreactors before Day 90 (*P* = 0.06–0.94 by a two‐tailed *t*‐test on each time point) (Figure 5B). On Day 90, the mean weighted community determinism of treatment bioreactors was significantly higher than that of the controls (*P* = 0.02 by a two‐tailed *t*‐test). On Day 97, which was before the collapse of treatment bioreactors, both the weighted determinism and the unweighted community determinism were substantially higher in the treatment bioreactors than the controls (*P* = 0.004 for weighted community determinism and *P* = 0.04 for unweighted community determinism by a two‐tailed *t*‐test) (Figure 5B), indicating stronger selection in the treatment bioreactors.

## DISCUSSION

Untangling ecological processes controlling community dynamics is a major challenge in microbial ecology, primarily due to the lack of an appropriate theoretical framework and long‐term time‐series data sets^13^, ^49^. With recent advances of genomics technology, massive longitudinal data can be rapidly obtained across different environmental conditions^50^, which offers great opportunities for testing microbial ecological theories^15^, ^51^. Here, we described a novel process model‐based framework to quantitatively assess the assembly mechanisms controlling community dynamics. Different from statistical approaches such as VPA^52^, ^53^ and null model‐based methods^15^, ^51^, ^54^, ^55^, the process models are mechanistically developed to enable the prediction of community dynamics and their underlying mechanisms. Our analyses demonstrate that this framework could discern the relative importance of deterministic processes (immigration, resource competition) and the stochastic process of drift in driving taxa and community dynamics. The developed framework represents a significant advance in reconciling both niche and neutral theories for predicting community dynamics and underlying mechanisms toward predictive microbial ecology, the ultimate goal in this field.

Microbial rarity can result from both stochastic and deterministic processes^56^. For instance, low local abundance can emerge by stochastic population fluctuations. A recently immigrated taxon might also be rare when it first enters a new community. Niche processes, including abiotic and biotic factors, can play crucial roles in driving taxon rarity. Rare biosphere members can be ascribed to narrow niche breadth, thus remaining generally inactive and at low density in most conditions but becoming dominant when favorable conditions arise^57^, ^58^, which is best illustrated by the extreme case of microbial dormancy. An alternative is the competition–colonization trade‐off hypothesis, which is rooted in the classic niche‐based ecology predicting that taxa with low competitive ability may remain rare rather than become extinct due to the advantage in immigration and colonization^59^, ^60^. Since microbial dynamics are very fast, competitive exclusion may not have sufficient time to play out^61^. Our study suggested that immigration played important roles in driving community dynamics, especially for rare taxa (Figure 4). Rare microbial populations were shown to have the best fit to the neutral model in both control and treatment bioreactors (Figure 2A), indicating a dominant role of immigration and drift in shaping rare taxa dynamics, consistent with the observation that ecological drift was pronounced for rare planktonic eukaryotes^62^. Further, the estimated relative immigration rate was higher for rare taxa than abundant taxa (Figure 4A). This also supports the competition–colonization trade‐off hypothesis that rare taxa are recruited mainly through immigration^58^, ^63^. It was noted that the determinism of rare taxa was higher than abundant taxa (Figure 5A), which could be explained by their immigration rate. Higher immigration rate of a taxon would result in less variations in its relative abundances, as the taxon tends to return to its correspondent relative abundance in the metacommunity^12^, that is, higher determinism of taxa dynamics. In contrast, taxa with low immigration rates are less affected by the metacommunity, which may be subject to larger effects of local drift and result in more variations in their relative abundances.

Deterministic processes of resource competition might play an important role in shaping the dynamics of abundant taxa in treatment bioreactors, consistent with the resource‐related theory. The resource‐ratio theory successfully explained the “paradox of enrichment” in sludge bioreactors, that is, higher resource levels of nitrogen and oxygen initially increased and then decreased the diversity of the ammonia‐oxidizing bacteria^26^, as a result of competition among multiple taxa with different resource‐ratio requirements. A modified consumer–resource model to include nonspecific cross‐feeding interactions explained experimental results that many microbial taxa could co‐exist in a single‐resource environment^27^. Exploitative competition, rooted in the consumer–resource model, significantly contributed to abundant taxa dynamics in the disturbed environment (Figure 2A), possibly because increases in resources stimulated the competition among abundant microbial populations. As a result, the determinism at the community level was significantly higher in the treatment bioreactors as compared to the controls (Figure 5B).

The estimated competition strengths showed a stronger phylogenetic signal in the treatment than control bioreactors (Figure 3A). Temporal dynamics patterns of closely related ESVs were more similar in treatment bioreactors than the controls (Figure 3B), resonating with the physics‐based theory that views microbial community as a fully disordered background with unstructured individuals (i.e., behaviors of individuals are not clustered by their taxonomic identity)^64^, and that imposing disturbance will order the disordered individuals based on traits, resulting in ecological clusters that are disturbance dependent.

Understanding the mechanisms underlying community assembly is important not only to ecologists but also to practitioners. The relative importance of deterministic vs stochastic processes in controlling microbial community assembly has increasingly attracted interest in the last several years^4^. Since the treatment reactors were operated under fluctuating resource levels^45^, the microbial communities in treatment reactors appear more filtered compared to the control reactors under stable operating conditions, resulting in higher determinism. Our findings that deterministic processes are more important for controlling the taxa and community dynamics in the treatment reactors (Figure 5) are highly consistent with this expectation. In addition, the knowledge learned in this study could help environmental engineers maintain microbial systems for desired functions. For example, the neutral model could predict how taxa fluctuate in the control bioreactors (exemplified in Figure 4B). Given its simplicity, the neutral model could be useful in long‐term monitoring of stable systems such as wastewater treatment plants and human guts. The deviation of certain taxa from the predicated range may signify abnormal conditions of the system. Also, the increase of community determinism could provide early warnings for system functional instability, as exemplified by the treatment bioreactors before system collapse (Figure 5B). The relative competition strengths inferred from the consumer–resource model or the combined model can be used to identify functionally important taxa. Since abundant microbial populations play significant roles in biogeochemical cycling in ecosystems^65^, it is interesting to examine how changes in such functionally important taxa would affect resources such as the carbon pool by considering the coupled dynamics of resource and consumer under the framework of ecological stoichiometry^66^.

In this study, we demonstrated the applicability of the novel modeling framework in representing the bacterial community dynamics of anaerobic bioreactors. Given its mechanistic basis, the framework developed in this study is expected to be potentially applicable in other ecosystems such as soils, oceans, and guts and also to other organisms such as eukaryotic microorganisms and plants. We expect the neutral model to be an appropriate tool for modeling taxa dynamics in relatively stable environments such as human guts, while the combined model might be better for the abundant taxa in ecosystems with fluctuating resource levels such as soils. However, the performance of different models as well as the driving forces governing taxa dynamics in different ecosystems remain to be tested. It is also noted that these models possess certain limitations. For example, the resource level is assumed to linearly affect the taxa growth in the consumer–resource model and the combined model, which may not capture the complicated interaction between consumers and resources in nature. In addition, to achieve reliable parameter estimation for the SDE‐based models, extensive time‐series data of high frequency and duration must be collected, which often entails significant time and effort.

## MATERIALS AND METHODS

### Mathematical framework

#### Consumer–resource model

Because of its mathematically tractable form, MacArthur's consumer–resource model^38^ has a strong impact on the theory of exploitative competition^39^. In this study, we used the following equation^27^, ^40^ for its simplicity to describe the consumer–resource interaction of taxon i:

(1) $$\frac{dN_{i}}{\mathrm{dt}}=\left(\sum_{j}{b_{\mathrm{ij}}C}_{\mathrm{ij}}R_{j}-m_{i}\right)N_{i}$$

where $N_{i}$ is the absolute abundance (i.e., population density, population per unit area) of Taxon i and $R_{j}$ is the availability of Resource j. $C_{\mathrm{ij}}$ is the rate at which taxon i consumes Resource j, while the quality factor, $b_{\mathrm{ij}}$, represents taxon i's ability to convert the consumed resource into its biomass. Thus, their product, ${b_{\mathrm{ij}}C}_{\mathrm{ij}}$, can represent the competition strength of taxon i over Resource j. $m_{i}$ represents the minimum maintenance cost.

The community size, $N_{T}=\sum_{i=1}^{n}N_{i}$, is implicitly a function of time. For typical microbial community data, $N_{T}$ is not available. Rather, the relative abundances and the ratios between taxa abundances can be inferred from the compositional data sets^41^. We can choose a reference taxon r, and take the ratio of focal taxon and the reference taxon. Let $Z_{i}=\log\frac{N_{i}}{N_{r}}$ be the log ratio of taxon i to the reference taxon r. Based on Equation (1), we have

(2) $$\frac{d{\log(N}_{i})}{\mathrm{dt}}=\frac{dN_{i}}{N_{i}\mathrm{dt}}=\sum_{j}{b_{\mathrm{ij}}C}_{\mathrm{ij}}R_{j}-m_{i}$$

Thus,

(3) $$\begin{array}{ccl} \frac{dZ_{i}}{dt} & = & \frac{d\log\frac{N_{i}}{N_{r}}}{dt}=\frac{d{\log(N}_{i})}{dt}-\frac{d{\log(N}_{r})}{dt}\\ & = & \sum_{j}\left({b_{ij}C}_{ij}-{b_{rj}C}_{rj}\right)R_{j}-\left(m_{i}-m_{r}\right) \end{array}$$

In this study of the bioreactor data set, the availability of resource $R_{j}$ is represented by a single variable, the volatile solids (VS), in the bioreactors. $R_{j}$ could be represented by other resources when applying this model in other systems. These variables are known at discrete time points. Further, Equation (3) can be expressed as a simple linear model

(4) $$\frac{dZ_{i}}{\mathrm{dt}}=k_{0}+\sum_{j}k_{1,j}Y_{1,j}$$

where $k_{0}=-\left(m_{i}-m_{r}\right)$ represents the relative maintenance cost of taxon i as compared to the reference taxon, $k_{1,j}={b_{\mathrm{ij}}C}_{\mathrm{ij}}-{b_{\mathrm{rj}}C}_{\mathrm{rj}}$ represents the relative competition strength of taxon i over resource $R_{j}$, and $Y_{1,j}=R_{j}$. We can then estimate the parameters through a least‐squares regression analysis based on the measured variables at discrete time points.

#### The neutral model

In a neutral local community, when an individual dies, it is replaced by an immigrant of taxon *i* from a source community (i.e., regional species pool) with the probability $m_{i}$ or by regeneration from the local community with probability $1-m_{i}$. Under the neutral assumption, $m_{1}=m_{2}=\cdots =m$. We set the meantime for replacement of an individual to be *a* and define a scaled time *τ* = *t/a*. In a short time period ∆τ→0, we can expect only one replacement in the community. The species relative abundance Xin a neutral model follows a Wright–Fisher Process (WFP)^42^, ^43^, ^44^, which is defined by the Ito SDE:

(5) dX=λ(p−X)dτ+σ(X)dW

where p is the relative abundance of taxa in the metacommunity and $\lambda =N_{T}m$ is the product of local community size and taxon immigration probability, representing the relative rate of migration from the metacommunity into the local community. σ(X) is the instantaneous standard deviation of changes in X per unit time. dW is a standard Wiener process term. The quadratic covariation between taxa is given by $\sum \;=\frac{1}{2}\sigma \sigma^{T}$, where^42^, ^43^, ^44^

$$\sum_{ij}=\left\{\begin{array}{lc} X_{i}\left(1-X_{i}\right) & i=j\\ -X_{i}X_{j} & i\neq j \end{array}\right.$$

The SDE for the focal taxon i is then defined as

(6) $$dX_{i}=\lambda \left(p_{i}-X_{i}\right)\;d\tau +\sigma \left(X_{i}\right)dW_{i}=\underset{\mathrm{deterministic}}{\underbrace{\lambda \left(p_{i}-X_{i}\right)\;d\tau }}+\underset{\mathrm{stochastic}}{\underbrace{\sqrt{2X_{i}\left(1-X_{i}\right)}dW_{i}}}$$

where $X_{i}$ is the relative abundance of taxon i, that is, $X_{i}=\frac{N_{i}}{N_{T}}$. $dW_{i}$ is a standard Wiener process term following the standard normal distribution *N* (0,1). The first term on the right‐hand side of Equation (6) represents the expect change of $X_{i}$, which is a deterministic term; the second term represents the variation of change, which is a stochastic term.

The covariation between taxon i and taxon j (i≠j) is $E\left[\left(dX_{i}-E\left(dX_{i}\right)\right)\left(dX_{j}-E\left(dX_{j}\right)\right)\right]=E\left(\sqrt{{2X}_{i}\left(1-X_{i}\right)}dW_{i}\times \sqrt{{2X}_{j}\left(1-X_{j}\right)}dW_{j}\right)$, which equals to $-2X_{i}X_{j}$. This gives us the covariance between the two Wiener processes $dW_{i}$ and $dW_{j}$:

(7) $$\rho =E\left(dW_{i}dW_{j}\right)=-\sqrt{\frac{X_{i}X_{j}}{\left(1-X_{i}\right)\left(1-X_{j}\right)}}.$$

We can take the log‐ratio transformation as $Z_{i}=\log\frac{N_{i}}{N_{r}}=\log\frac{N_{i}/N_{T}}{N_{r}/N_{T}}=\log\frac{X_{i}}{X_{r}}$. Since both $X_{i}$ and $X_{r}$ follow the SDE (Equation 6), the SDE of $Z_{i}$ is derived based on Ito's lemma:

$$\begin{array}{ccl} dZ_{i} & = & \left[\frac{\partial Z_{i}}{\partial X_{i}}\lambda_{i}\left(p_{i}-X_{i}\right)+\frac{\partial Z_{i}}{\partial X_{r}}\lambda_{r}\left(p_{r}-X_{r}\right)+\frac{\partial Z_{i}}{\partial t}\right]d\tau \\ & & +\;\;\left[\frac{1}{2}\frac{\partial^{2}Z_{i}}{\partial X_{i}^{2}}\sigma^{2}\left(X_{i}\right)+\frac{\partial^{2}Z_{i}}{\partial X_{i}\partial X_{r}}\sigma \left(X_{i}\right)\sigma \left(X_{r}\right)\rho +\frac{1}{2}\frac{\partial^{2}Z_{i}}{\partial X_{r}^{2}}\sigma^{2}\left(X_{r}\right)\right]d\tau \\ & & +\;\frac{\partial Z_{i}}{\partial X_{i}}\sigma \left(X_{i}\right)dW_{i}+\frac{\partial Z_{i}}{\partial X_{r}}\sigma \left(X_{r}\right)dW_{r} \end{array}$$

That is,

(8) $$\begin{array}{ccc} dZ_{i} & = & \left[\frac{\lambda_{i}p_{i}-1}{X_{i}}-\frac{\lambda_{r}p_{r}-1}{X_{r}}+\lambda_{r}-\lambda_{i}\right]d\tau \\ & & +\;\sqrt{\frac{2\left(1-X_{i}\right)}{X_{i}}}dW_{i}-\sqrt{\frac{2\left(1-X_{r}\right)}{X_{r}}}dW_{r} \end{array}$$

Given that *τ* = *t/a*, and the covariance between $dW_{i}$ and $dW_{r}$ (Equation 7), the above equation (Equation 8) can be written as a SDE:

(9) $$dZ_{i}=\underset{deterministic}{\underset{︸}{\frac{1}{a}\left[\frac{\lambda_{i}p_{i}-1}{X_{i}}-\frac{\lambda_{r}p_{r}-1}{X_{r}}+\lambda_{r}-\lambda_{i}\right]dt}}+\underset{stochastic}{\underset{︸}{\sqrt{\frac{2}{aX_{i}}+\frac{2}{aX_{r}}}dW_{t}}}$$

where $dW_{t}$ is a Wiener process term, which follows a normal distribution *N* (0, *dt*). Further, Equation (9) can be expressed as a simple linear model,

(10) $$\frac{dZ_{i}}{\mathrm{dt}}=k_{0}+k_{2}Y_{2}+k_{3}Y_{3}+\;\varepsilon$$

where $k_{0}=\frac{\lambda_{r}-\lambda_{i}}{a}$, $k_{2}=\frac{\lambda_{i}p_{i}-1}{a}$, $Y_{2}=\frac{1}{X_{i}}$, $k_{3}=-\frac{\lambda_{r}p_{r}-1}{a}$, $Y_{3}=\frac{1}{X_{r}}$, and ε is an error term given by $\varepsilon =\sqrt{\frac{2}{{\mathrm{aX}}_{i}}+\frac{2}{{\mathrm{aX}}_{r}}}\frac{{\mathrm{dW}}_{t}}{\mathrm{dt}}$. The parameters can be estimated through a weighted least‐squares regression analysis, in which the weights are $\frac{\mathrm{dt}}{\frac{2}{X_{i}}+\frac{2}{X_{r}}}$. The weighted errors should be normally distributed and the standard residual error of the linear regression model should be $\sqrt{\frac{1}{a}}$. We then estimate the parameter product, $\lambda_{i}p_{i}$, based on the coefficient of variable $Y_{2}$. Further, $p_{i}$ can be estimated as the mean relative abundance of taxon i and $\lambda_{i}$ can be derived by dividing the estimated $\lambda_{i}p_{i}$ to $p_{i}$.

#### The combined model

A combined model of taxon dynamics was further developed to include both exploitative competition and neutral factors. The term of “relative growth” (can be positive or negative) caused by the resource consumption (Equation 3) is added to the deterministic part of the SDE (Equation 9) without change, since it is purely deterministic, which would not bring in any uncertainty. The combined model is thus given by:

(11) $$dZ_{i}=\underset{\mathrm{deterministic}}{\underbrace{\left[\frac{\lambda_{i}p_{i}-1}{aX_{i}}-\frac{\lambda_{r}p_{r}-1}{aX_{r}}+\frac{\lambda_{r}}{a}-\frac{\lambda_{i}}{a}+\sum_{j}\left(b_{ij}C_{ij}-b_{rj}C_{rj}\right)R_{j}-\left(m_{i}-m_{r}\right)\right]dt}}+\underset{\mathrm{stochastic}}{\underbrace{\sqrt{\frac{2}{aX_{i}}+\frac{2}{aX_{r}}}dW_{t}}}$$

Further, Equation (11) can be expressed as a simple linear model,

(12) $$\frac{dZ_{i}}{\mathrm{dt}}=k_{0}+\sum_{j}k_{1,j}Y_{1,j}+k_{2}Y_{2}+k_{3}Y_{3}+\varepsilon$$

where $Z_{i}=\log\frac{X_{i}}{X_{r}}$ is the log ratio of the relative abundance of taxon i to the reference taxon r. $k_{0}=\frac{\lambda_{r}}{a}-\frac{\lambda_{i}}{a}+m_{r}-m_{i}$, $k_{1,j}={b_{\mathrm{ij}}C}_{\mathrm{ij}}-{b_{\mathrm{rj}}C}_{\mathrm{rj}}$ represent the relative competition strength of taxon i on resource $R_{j}$, and $Y_{1,j}=R_{j}$. $k_{2}=\frac{\lambda_{i}p_{i}-1}{a}$, $Y_{2}=\frac{1}{X_{i}}$, $k_{3}=-\frac{\lambda_{r}p_{r}-1}{a}$, $Y_{3}=\frac{1}{X_{r}}$, and ε is an error term given by $\varepsilon =\sqrt{\frac{2}{{\mathrm{aX}}_{i}}+\frac{2}{{\mathrm{aX}}_{r}}}\frac{dW_{t}}{\mathrm{dt}}$. The parameters can be estimated through a weighted least‐squares regression analysis, in which the weights are $\frac{\mathrm{dt}}{\frac{2}{X_{i}}+\frac{2}{X_{r}}}$. The weighted errors should be normally distributed and the standard residual error of the linear regression model should be $\sqrt{\frac{1}{a}}$. $p_{i}$ can be estimated as the mean relative abundance of taxon i. We can estimate the parameters $k_{0}$, $k_{1}$, $k_{2}$, and $k_{3}$ in the linear model, by which the model parameters ${b_{\mathrm{ij}}C}_{\mathrm{ij}}-{b_{\mathrm{rj}}C}_{\mathrm{rj}}$, $\lambda_{i}$, and $p_{i}$ can be further derived.

#### Determinism

The SDE of the combined model (Equation 11) can be written as

$$dZ=\underset{\mathrm{deterministic}}{\underbrace{\mu d\tau }}+\underset{\mathrm{stochastic}}{\underbrace{\sigma dW}}$$

where μ is the expected change of Z per unit time and σ is the instantaneous standard deviation of changes in Z per unit time. dW is a standard Wiener process term. We define taxa determinism as the inverse of the variation coefficient, that is,

(13) $$\mathrm{determinism}=\frac{\mu }{\sigma }$$

After parameter estimation using weighted least‐squares regression analysis, the taxa determinism can be calculated for each taxon at each time point based on Equation (13). For the combined model, the determinism of taxon i can be calculated based on parameters of the linear model Equation (12):

(14) $$\mathrm{determinism}=\frac{\left(k_{0}+\sum_{j}k_{1,j}R_{j}+\frac{k_{2}}{X_{i}}+\frac{k_{3}}{X_{r}}\right)\times a}{\sqrt{\frac{2}{X_{i}}+\frac{2}{X_{r}}}}$$

Note that the stochasticity is calculated on the scaled time unit τ. Then, the community‐level determinism is calculated as the mean determinism among taxa, either weighted by the relative abundance of each taxon (weighted determinism) or not (unweighted determinism).

### Anaerobic bioreactor operation and 16S rRNA gene sequencing

The operation of anaerobic bioreactors, biomass sampling, and chemical analyses were processed as previously described^45^. In brief, two sets of triplicated, continuous anaerobic bioreactors (i.e., the control bioreactors C1, C2, and C3 and the treatment bioreactors D1, D2, and D3) were operated at 35°C and fed at 4‐h intervals, each with a working volume of 3.6 l. The control bioreactors were fed with dairy manure at a constant rate and continuously operated for 501 days, which showed a stable organic matter level (Figure S1A). The treatment bioreactors were operated for 100 days before they collapsed by supplementing incremental poultry waste, resulting in higher ammonia toxicity (Figure S1B). Sludge samples were generally taken every 3–10 days from each bioreactor, resulting in 53‐time points for control and 11‐time points for treatment bioreactors.

DNA extraction and 16S rRNA gene sequencing were processed as previously described^45^. In brief, biomass samples were suspended in 630 μl of DNA‐extraction buffer and treated with a lysozyme mixture (60 μl, 37°C, 60 min), a protease mixture (60 μl, 37°C, 30 min), and 20% sodium dodecyl sulfate (80 μl, 37°C, 90 min). The treated sample suspension was then extracted using phenol–chloroform–isoamyl alcohol (25:24:1) at 65°C for 20 min, followed by extraction with chloroform–isoamyl alcohol (24:1) to obtain a supernatant. Further, DNA extract was combined with 0.6 volume of isopropanol and stored overnight at 4°C; DNA was obtained through centrifugation, followed by washing with 70% cold ethanol, drying, and resuspension in nuclease‐free water. The purity and concentration of DNA were subsequently assessed utilizing a NanoDrop spectrophotometer (NanoDrop Technologies Inc.). The V4 region of the microbial 16S rRNA gene was amplified by primer pairs of 515 F and 806 R^46^. PCR amplicon sequencing was conducted on the MiSeq Illumina platform at the Institute for Environmental Genomics (IEG), University of Oklahoma. Sequences were processed to generate exact ESVs by UNOISE3^47^ at the 100% sequence similarity threshold. ESVs with fewer than eight reads were removed using the default “‐minsize” values. Taxonomy was assigned with a confidence cutoff of 50% using the RDP classifier^48^. The reference taxon was then chosen as the one with the top frequency and relative abundance, which was ESV1 detected at all time points.

Since there were only 11‐time points for each treatment bioreactor, we combined the time series of the triplicate bioreactors together to improve the reliability of model fitting. For example, if the dependent variable (as for Equations 4, 10, and 12) of one taxon in treatment bioreactor D1 is ${\left(\frac{{\mathrm{dZ}}_{i}}{\mathrm{dt}}\right)}_{D1}=\left[{\left(\frac{{z_{i,t2}-z}_{i,t1}}{t2-t1}\right)}_{D1,1},\ldots ,{\left(\frac{{z_{i,t11}-z}_{i,t10)}}{t11-t10}\right)}_{D1,10}\right]$, the dependent variable of this taxon in D2 is ${\left(\frac{{\mathrm{dZ}}_{i}}{\mathrm{dt}}\right)}_{D2}=\left[{\left(\frac{{z_{i,t2}-z}_{i,t1}}{t2-t1}\right)}_{D2,1},\ldots ,{\left(\frac{{z_{i,t11}-z}_{i,t10)}}{t11-t10}\right)}_{D2,10}\right]$ and that in D3 is ${\left(\frac{{\mathrm{dZ}}_{i}}{\mathrm{dt}}\right)}_{D3}=\left[{\left(\frac{{z_{i,t2}-z}_{i,t1}}{t2-t1}\right)}_{D3,1},\ldots ,{\left(\frac{{z_{i,t11}-z}_{i,t10}}{t11-t10}\right)}_{D3,10}\right]$, then the dependent variable for the combined time series is ${\left(\frac{{\mathrm{dZ}}_{i}}{\mathrm{dt}}\right)}_{D}=\left[{\left(\frac{{\mathrm{dZ}}_{i}}{\mathrm{dt}}\right)}_{D1},{\left(\frac{{\mathrm{dZ}}_{i}}{\mathrm{dt}}\right)}_{D2},{\left(\frac{{\mathrm{dZ}}_{i}}{\mathrm{dt}}\right)}_{D3}\right]=\left[\begin{array}{l} {\left(\frac{{z_{i,t2}-z}_{i,t1}}{t2-t1}\right)}_{D1,1},\ldots ,{\left(\frac{{z_{i,t11}-z}_{i,t10}}{t11-t10}\right)}_{D1,10},{\left(\frac{{z_{i,t2}-z}_{i,t1}}{t2-t1}\right)}_{D2,1},\ldots ,\\ {\left(\frac{{z_{i,t11}-z}_{i,t10}}{t11-t10}\right)}_{D2,10},{\left(\frac{{z_{i,t2}-z}_{i,t1}}{t2-t1}\right)}_{D3,1},\ldots ,{\left(\frac{{z_{i,t11}-z}_{i,t10}}{t11-t10}\right)}_{D3,10} \end{array}\right]$. Similarly, the independent variables can be combined in the same way. The combined dependent and independent variables for the treatment bioreactors were then used for the linear regression analyses based on the least‐squares method. We note that this is not a standard way to apply the model fitting for common time‐series data. Yet, this combination method may provide an option for replicated time series. In fact, fluctuations in microbial community compositions were highly consistent for the three replicated treatment bioreactors (Figure S1C), which enabled us to test the dynamical pattern of microbial taxa based on the combined time series.

## AUTHOR CONTRIBUTIONS

**Linwei Wu**: Conceptualization (lead); formal analysis (lead); methodology (lead); writing—original draft (lead). **Yunfeng Yang**: Conceptualization (equal); supervision (equal); writing—review and editing (equal). **Daliang Ning**: Methodology (equal); project administration (equal). **Qun Gao**: Writing—review and editing (equal). **Huaqun Yin**: Data curation (equal). **Naija Xiao**: Methodology (equal). **Benjamin Y. Zhou**: Formal analysis (equal). **Si Chen**: Data curation (equal). **Qiang He**: Data curation (equal); project administration (equal). **Jizhong Zhou**: Conceptualization (equal); funding acquisition (equal); project administration (equal); supervision (equal); writing—review and editing (equal).

## ETHICS STATEMENT

No animals and human were involved in this study.

## CONFLICT OF INTERESTS

The authors declare no conflict of interests.

## Supporting information

Supporting information.

## Data Availability

Sequence data are accessible in the GenBank database under the accession number SRP070491. R codes on the modeling and statistical analyses are available at https://github.com/Linwei-Wu/species_dynamics_models.
