# Supplementary material for: Assessing mechanisms for microbial taxa and community dynamics using process models
Source: mLife. 2023 Sep 12;2(3):239–52. doi: 10.1002/mlf2.12076 (PMC10989933; doi:10.1002/mlf2.12076)
Supplement: Supplementary file 1 — Supporting information. [file MLF2-2-239-s001.docx]

Supplementary Information for

**Assessing mechanisms for microbial taxa and community dynamics using process models**

Linwei Wu, Yunfeng Yang, Daliang Ning, Qun Gao, Huaqun Yin, Naija Xiao, Benjamin Y. Zhou, Si Chen, Qiang He, and Jizhong Zhou

Correspondence: Linwei Wu

Email: linwei.wu@pku.edu.cn

**This PDF file includes:**

Table S1 to S2

Figures S1 to S5

**Table. S1.** **The number and proportions of abundant, moderate and rara taxa in control or treatment reactors for model fitting.** There’s only one set of data for treatment reactors since we combined the three replicated time-series together for model fitting.

| Reactors | Rare taxa | Moderate taxa | Abundant taxa | Total taxa for modeling |
| --- | --- | --- | --- | --- |
| C1 | 1388 (62.5%) | 701 (31.6%) | 132 (5.9%) | 2221 |
| C2 | 1377 (62.6%) | 690 (31.4%) | 131 (6.0%) | 2198 |
| C3 | 1451 (63.9%) | 685 (30.7%) | 134 (5.9%) | 2270 |
| Treatment | 932 (52.7%) | 697 (39.4%) | 139 (7.9%) | 1768 |

**Table S2. Summary of the estimated** ${\boldsymbol{b}_{\boldsymbol{i}}\boldsymbol{C}}_{\boldsymbol{i}}\boldsymbol{-}{\boldsymbol{b}_{\boldsymbol{r}}\boldsymbol{C}}_{\boldsymbol{r}}$ **representing the relative competition strength for each taxon in the treatment and control bioreactors.** Since at least six data points are required for model fitting, taxa detected at less than six time points in control or treatment bioreactors were not subjected to model fitting and the parameters are indicated with ‘NA’ in the table.

*See the excel file.*


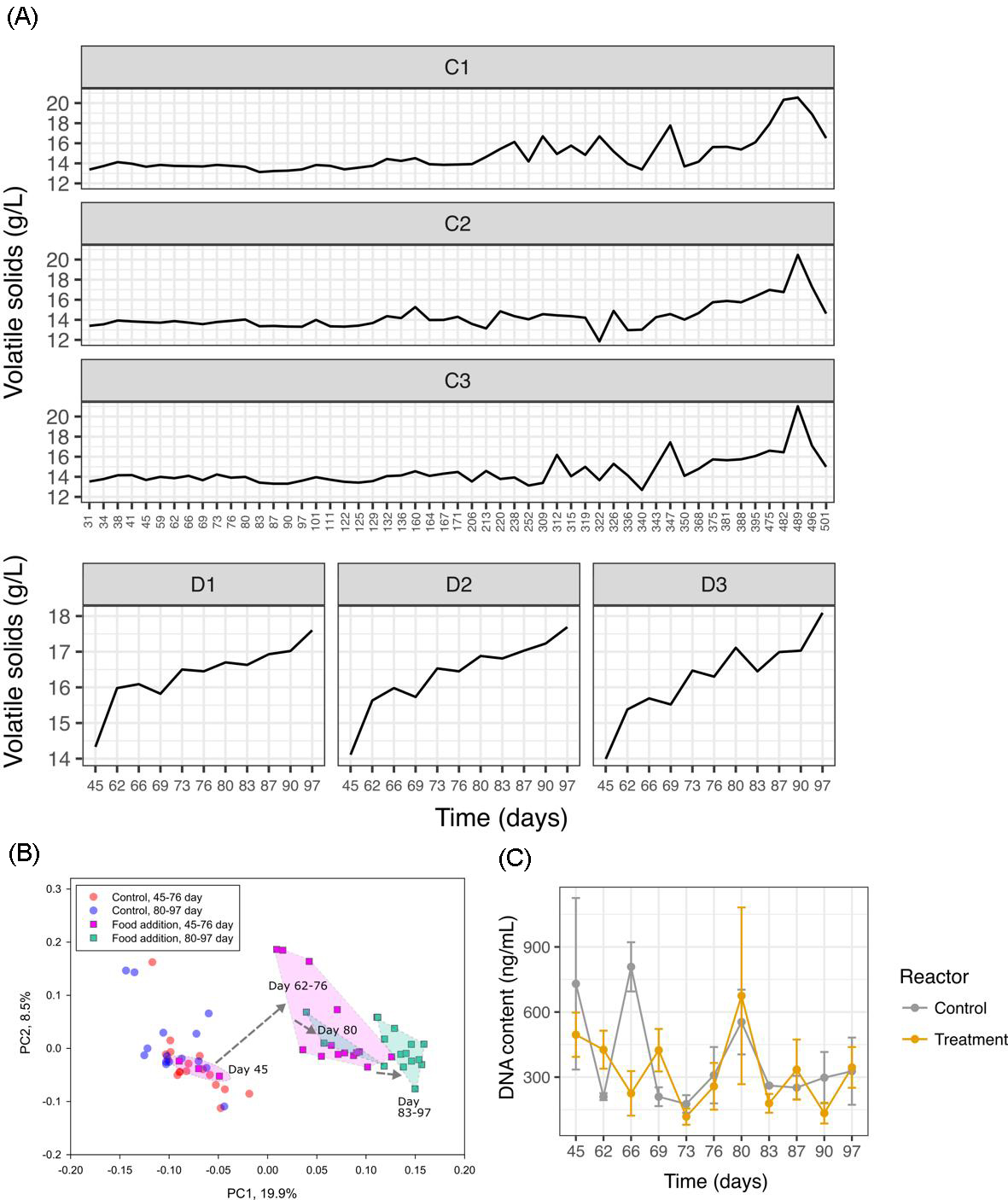


**Fig. S1.** **Background data on environmental resources and microbial communities**. (**A**)Volatile solids (VS) representing the organic matter level in control and treatment bioreactors. The VS levels were relatively stable in control reactors, especially during the time before day 300; while VS levels increased with time in treatment reactors. **(B)** Principal coordinates analysis (PcoA) showing obvious succession of microbial community compositions in treatment but not control reactors. **(C)** The DNA concentration, indicating microbial biomass, showed no obvious relationship with time despite certain fluctuations.


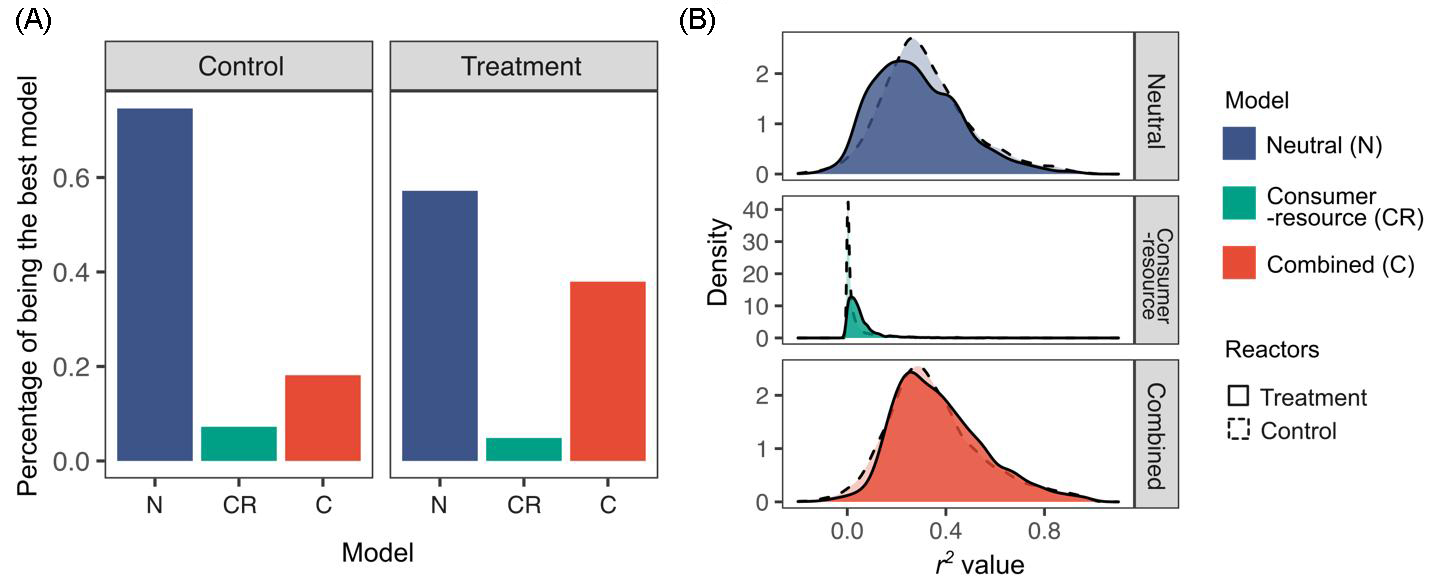


**Fig. S2.** **Comparing model fitting on all taxa between control and treatment bioreactors. (A)** For each taxon, we fitted the neutral model, the consumer-resource model and the combined model, and the best model for that taxon was determined as the one with the lowest Akaike information criteria (AIC) value. Percentages of each model being the best model for all taxa were shown. **(B)** The distribution of R^2^ values of the three models.

**
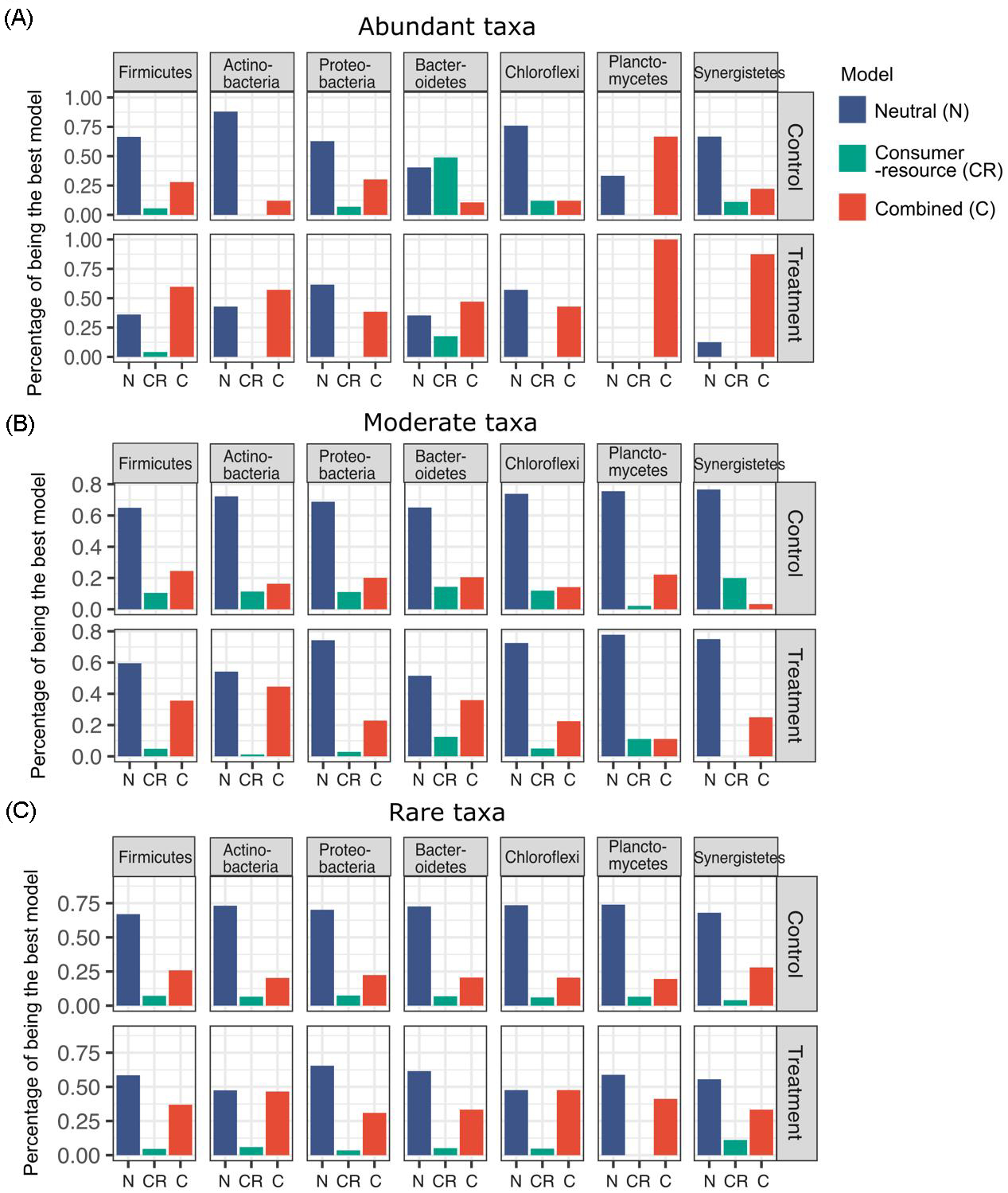
**

**Fig. S3.** **Comparing model fitting on time series of taxon relative abundance across different phyla.** For each taxon, we fitted the neutral model, the consumer-resource model and the combined model, and the best model for that taxon was determined as the one with the lowest Akaike information criteria (AIC) value. Percentages of each model being the best model for abundant (A), moderate (B) or rare taxa (C) within a phylum were shown.

**
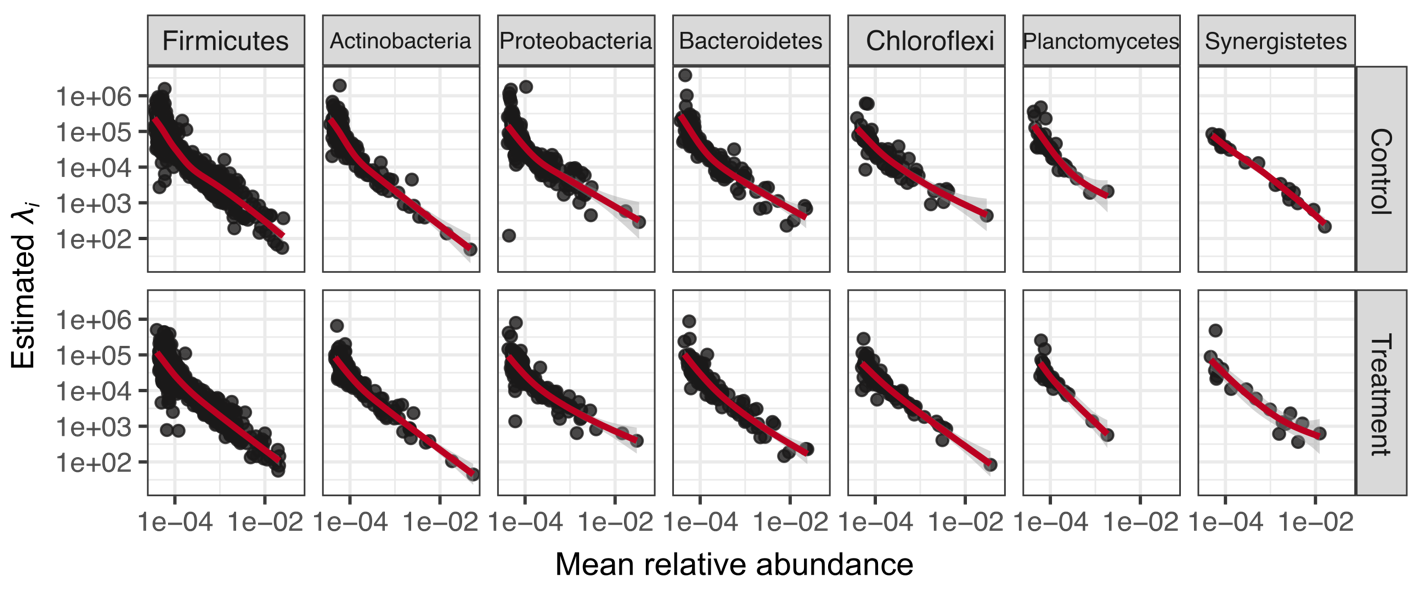
**

**Fig. S4. The estimated *λ_i_* from the neutral model versus the mean relative abundance of bacteria ESVs across phyla.** Within each phylum, the estimated *λ_i_* is negatively correlated with the relative abundance.

**
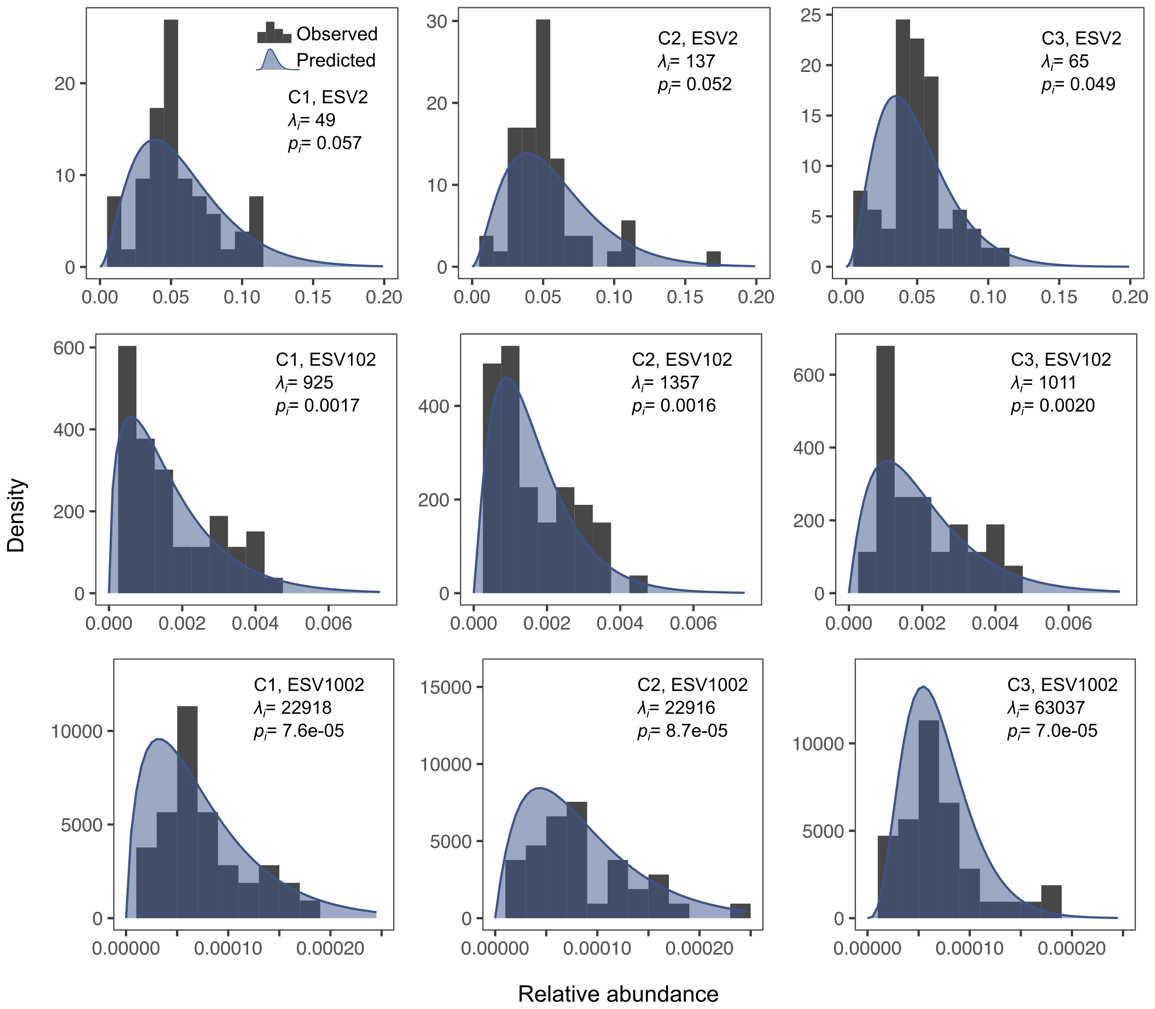
**

**Fig. S5. Prediction of the neutral model on the distribution of relative abundances of exemplified ESVs.** The grey histograms represent the observed value, and the blue shadow represent the model predictions using the parameters *λ_i_* and *p_i_* calibrated from the time series.
